# Supplementary material for: An allosteric site in the T-cell receptor Cβ domain plays a critical signalling role
Source: Nat Commun. 2017 May 16;8:15260. doi: 10.1038/ncomms15260 (PMC5440810; doi:10.1038/ncomms15260)
Supplement: Supplementary Information — Supplementary Tables and Supplementary Figures [file ncomms15260-s1.pdf]

Supplementary Table 1 | SPR Binding of WT and Asn133A mutant TCR to pMHC

**Expt. 1**	Steady State		Kinetics			
	K_D ± SE (M)	χ^2 (RU^2)	k_a ± SE ($M^{-1}s^{-1}$)	k_d ± SE (s^{-1})	K_D (M)	χ^2 (RU^2)
B4.2.3 WT	3.57×10^{-7} ± 5.3×10^{-9}	0.0048	6.71×10^{5} ± 1.3×10^{4}	0.212 ± 0.002	3.16×10^{-7}	0.16
N133A	4.98×10^{-7} ± 2.3×10^{-8}	0.038	5.55×10^{5} ± 8.8×10^{3}	0.204 ± 0.002	3.68×10^{-7}	0.092
**Expt. 2**						
B4.2.3 WT	4.10×10^{-7} ± 2.4×10^{-9}	0.0016	5.45×10^{5} ± 8.7×10^{3}	0.210 ± 0.002	3.85×10^{-7}	0.132
N133A	4.17×10^{-7} ± 7.6×10^{-9}	0.014	5.41×10^{5} ± 8.8×10^{3}	0.213 ± 0.002	3.95×10^{-7}	0.123
**Expt. 3**						
B4.2.3 WT	4.49×10^{-7} ± 1.5×10^{-8}	0.024	5.27×10^{5} ± 1.1×10^{4}	0.203 ± 0.002	3.86×10^{-7}	0.097
N133A	4.79×10^{-7} ± 1.9×10^{-8}	0.030	4.94×10^{5} ± 1.3×10^{4}	0.217 ± 0.003	4.38×10^{-7}	0.148
**Avg. of three expts.**						
	K_D (Mean ± SEM (M))		k_a (Mean ± SEM) ($M^{-1}s^{-1}$)	k_d (Mean ± SEM) (s^{-1})	K_D (Mean ± SEM) (M)	
B4.2.3 WT	4.05×10^{-7} ± 2.7×10^{-8}		5.81×10^{5} ± 4.5×10^{4}	0.208 ± 0.003	3.62×10^{-7} ± 2.3×10^{-8}	
N133A	4.65×10^{-7} ± 2.5×10^{-8}		5.30×10^{5} ± 1.8×10^{4}	0.211 ± 0.004	4.00×10^{-7} ± 2.0×10^{-8}	

SPR binding experiments were carried out as indicated in the legend to Supplemental Figure 7, and fit as described. Data for the three experiments were averaged as shown.

Supplementary Table 2 | Primer sequences for generating Ala mutants of selected TCR Cβ residues

Mutation	Primer sequence (5'-3')
Ser127Ala	TCCTTGTTTGAGCCAGCAAAAGCAGAGATTGCA
Glu130Ala	GAGCCATCAAAAGCAGCGATTGCAAACAAACAA
Asn133Ala	AAAGCAGAGATTGCAGCCAAACAAAAGGCTACC
Lys134Ala	GCAGAGATTGCAAACGCACAAAAGGCTACCCTC
Thr138Ala	AACAAACAAAAGGCTGCCCTCGTGTGCTTGGCC

DNA oligo primers used to prepare Ala mutants for functional assays as indicated in Fig. 7.

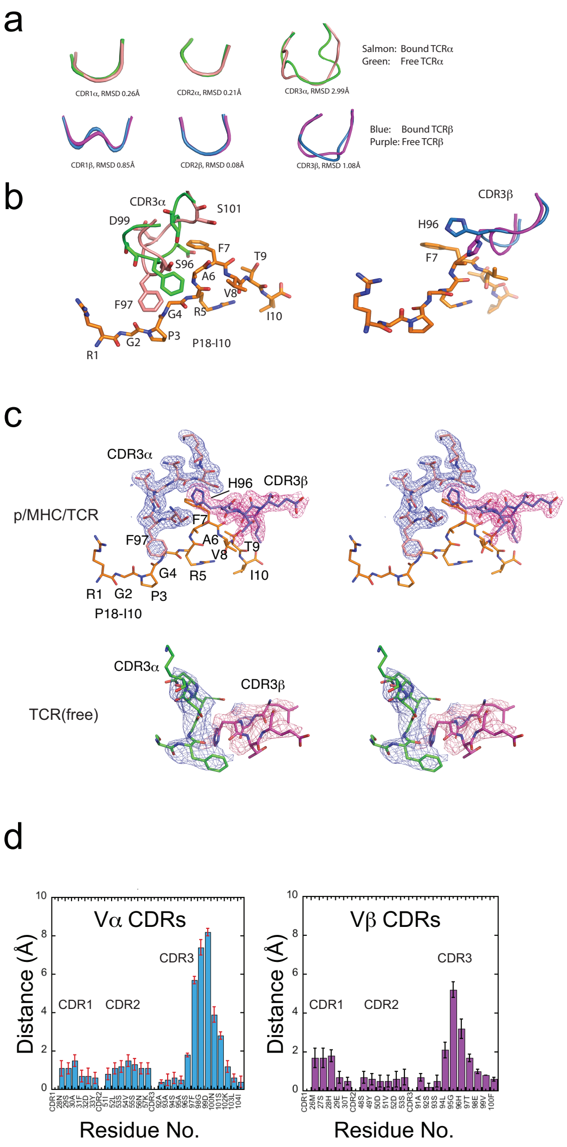

Supplementary Figure 1 | Superposition of unliganded and liganded TCR

a, Ribbon diagram representation of the superposition of the CDR loops of B4.2.3 TCR α-chain (top row) and β-chain (bottom row) in the free or P18-I10/H2-D^d bound states. The αβ TCR of the first heterodimer in the asymmetric unit was superposed on the B4.2.3/P18-I10/H2-D^d complex. The RMSD of the superposed C_α atoms is indicated. **b,** Structural diagram of the disposition of the B4.2.3 TCRα (left panel) or β (right panel) CDR3 loops before and after P18-I10/H2-D^d binding. P18-I10 is shown in stick representation. Colors are as indicated in **b**. **c,** Side-by-side stereo views of 2Fo-Fc electron density maps of CDR3α and β loops are shown with superposed models for P18-I10/H2-D^d/B4.2.3 TCR complex (upper panel) and for unliganded TCR (lower panel), contoured at 1.2σ for the complex and at 1.8σ for the unliganded TCR. **d,** Structural diversity of CDRs of unliganded B4.2.3 TCR. Each of the three TCR αβ heterodimers of the asymmetric unit of the unliganded structure was superposed on the TCR in the B4.2.3/P18-I10/H2-D^d complex. The difference in the distance of the indicated C_α atoms was tabulated, averaged and is plotted as the mean ± 1 standard deviation.

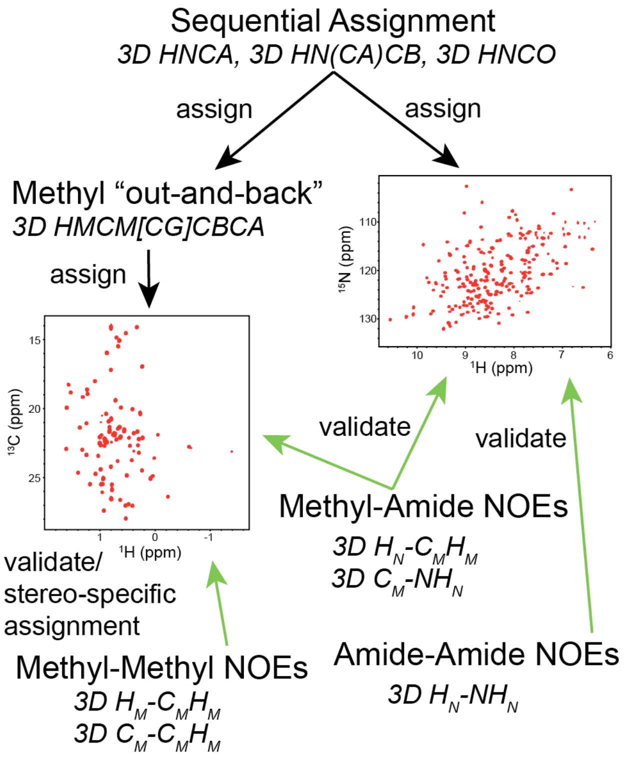

Supplementary Figure 2 | A multipronged approach for *de novo* assignment and cross-validation of backbone and sidechain methyl NMR resonances of β-chain labeled TCR
Backbone chemical shifts (^{15}N, ^{1}H$_{N}$, ^{13}C$_{\alpha}$, ^{13}C$_{\beta}$, ^{13}CO) were assigned through a combination of 3D HNCA, 3D HN(CA)CB and 3D HNCO triple-resonance experiments using a sequential assignment method (right black arrow, 2D ^{1}H-^{15}N TROSY-HSQC spectrum). Non-stereospecific sidechain methyl chemical shift assignments (Ile ^{13}Cδ1, Leu ^{13}Cδ1/^{13}Cδ2, Val ^{13}Cγ1/^{13}Cγ2) were obtained from a 3D HMCM[CG]CBCA methyl out-and-back experiment (left black arrow, 2D ^{1}H-^{13}C SOFAST HMQC spectrum), which also allowed for assignment of additional side-chain chemical shifts (Ile ^{13}Cγ1, Leu ^{13}Cγ). Ala ^{13}Cβ chemical shifts in the 2D ^{1}H-^{13}C SOFAST HMQC spectrum were assigned by comparison with the Ala ^{13}Cβ chemical shifts observed in the 3D HN(CA)CB experiment. Backbone amide chemical shift assignments were confirmed through the NOE patterns obtained in 3D H$_{N}$-NH$_{N}$ NOESY experiments, while sidechain methyl assignments were confirmed through NOEs obtained from 3D H$_{M}$-C$_{M}$H$_{M}$ and 3D C$_{M}$-C$_{M}$H$_{M}$ SOFAST NOESY experiments (green arrows). The methyl NOEs further allowed for disambiguation between the geminal Leu ^{13}Cδ1/^{13}Cδ2 and Val ^{13}Cγ1/^{13}Cγ2 resonances towards complete, stereospecific assignments (through close comparison of NOE cross-peak intensities with the corresponding distances in the 2.0 Å TCR crystal structure using a Python script in Sparky). Both amide and sidechain methyl assignments were cross-validated by obtaining methyl-to-amide NOEs from 3D H$_{N}$-C$_{M}$H$_{M}$ and 3D C$_{M}$-NH$_{N}$ SOFAST NOESY experiments (green arrows). The application of this procedure is detailed in Methods and outlined for two select regions of the molecule in **Supplementary Fig. 4, 5.** A final assignment completeness of 90% and 100% for non-Pro backbone amides and AILV sidechain methyls, respectively, is reported in the BMRB entry 26977.

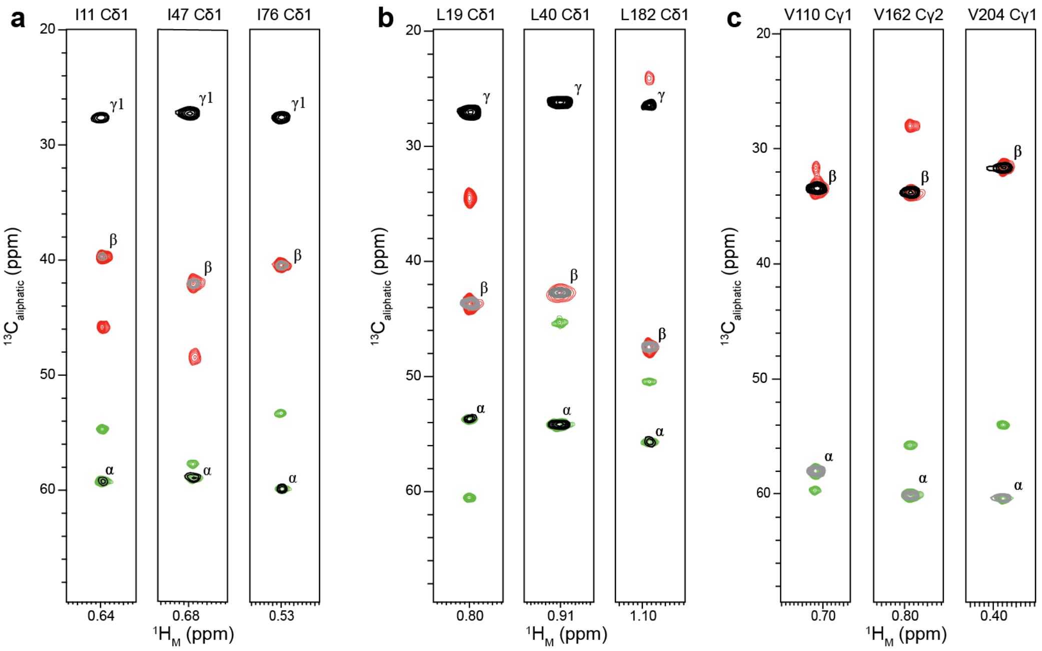

Supplementary Figure 3 | Assignment of NMR methyls in β-chain selectively ^{13}C methyl-labeled TCR

$^{13}C_{aliphatic}$-$^{1}H_M$ strips from a 3D HMCM[CG]CBCA experiment for **a,** Ile, **b,** Leu, and **c,** Val residues of methyl protonated ($^{13}CH_3$ δ1 only for Ile; non-stereospecific $^{13}CH_3$, $^{12}CD_3$ for Leu and Val) U-[^{15}N, ^{13}C, ^{2}H] labeled β chain B.4.2.3 TCR recorded at 800 MHz, 25ºC. Negative correlations (^{13}Cβ for Ile and Leu, ^{13}Cα for Val) are shown in grey, while positive correlations (^{13}Cγ1 for Ile, ^{13}Cγ for Leu, ^{13}Cβ for Val) are shown in black. The 3D HNCA (green) and 3D HN(CA)CB (red) strips for each residue are overlaid onto their respective 3D HMCM[CG]CBCA strip. Comparison of the of backbone 3D HNCA/3D HN(CA)CB and sidechain 3D HMCM[CG]CBCA correlation experiments allowed full assignment of methyl resonances.

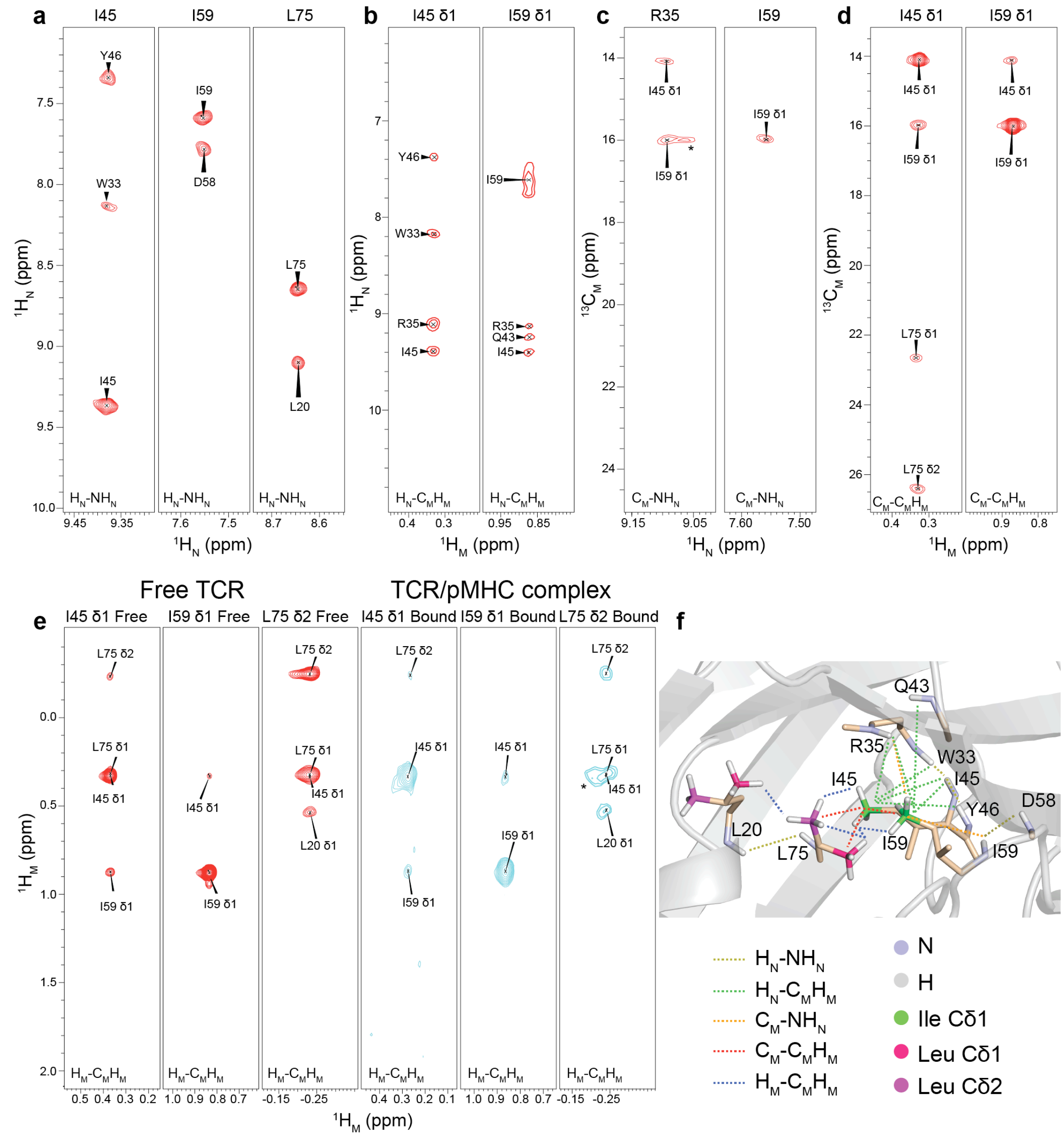

Supplementary Figure 4 | NOEs for β-chain TCR in the free and pMHC bound states

a, ^1H_N-^1H_N strips from a 3D H_N-NH_N NOESY experiments of residues I45, I59, and L75 in the free state. **b,** ^1H_N-^1H_M strips from a 3D H_N-C_MH_M SOFAST NOESY experiment of residues I45 δ1 and I59 δ1 in the free state. **c,** $^{13}C_M$-^1H_N strips from a 3D C_M-NH_N SOFAST NOESY experiment of residues R35 and I59 in the free state. **d,** $^{13}C_M$-^1H_M strips from a 3D C_M-C_MH_M SOFAST NOESY experiment of residues I45 δ1 and I59 δ1 in the free state. **e,** ^1H_M-^1H_M strips for I45 δ1, I59 δ1 and L75 δ2 from 3D H_M-C_MH_M SOFAST NOESY experiments on β-chain labeled B4.2.3 TCR in free (red) and P18-I10/H2-D^d bound (cyan) states. The peak indicated with an asterisk is centered on a different ^{13}C plane. **f,** Close up of residues I45, I59 and L75 on the β-chain from the X-ray structure of the B4.2.3 TCR showing NOEs between residues in the 3D H_N-NH_N (yellow dotted line), 3D H_N-C_MH_M (green dotted line), 3D C_M-NH_N (orange dotted line), 3D C_M-C_MH_M (red dotted line), and 3D H_M-C_MH_M (blue dotted line) NOESY experiments. Atoms are colored as followed: amide N (light blue stick), amide H (grey stick), Ile δ1 (green stick), Leu δ1 (light purple stick) and Leu δ2 (dark purple stick).

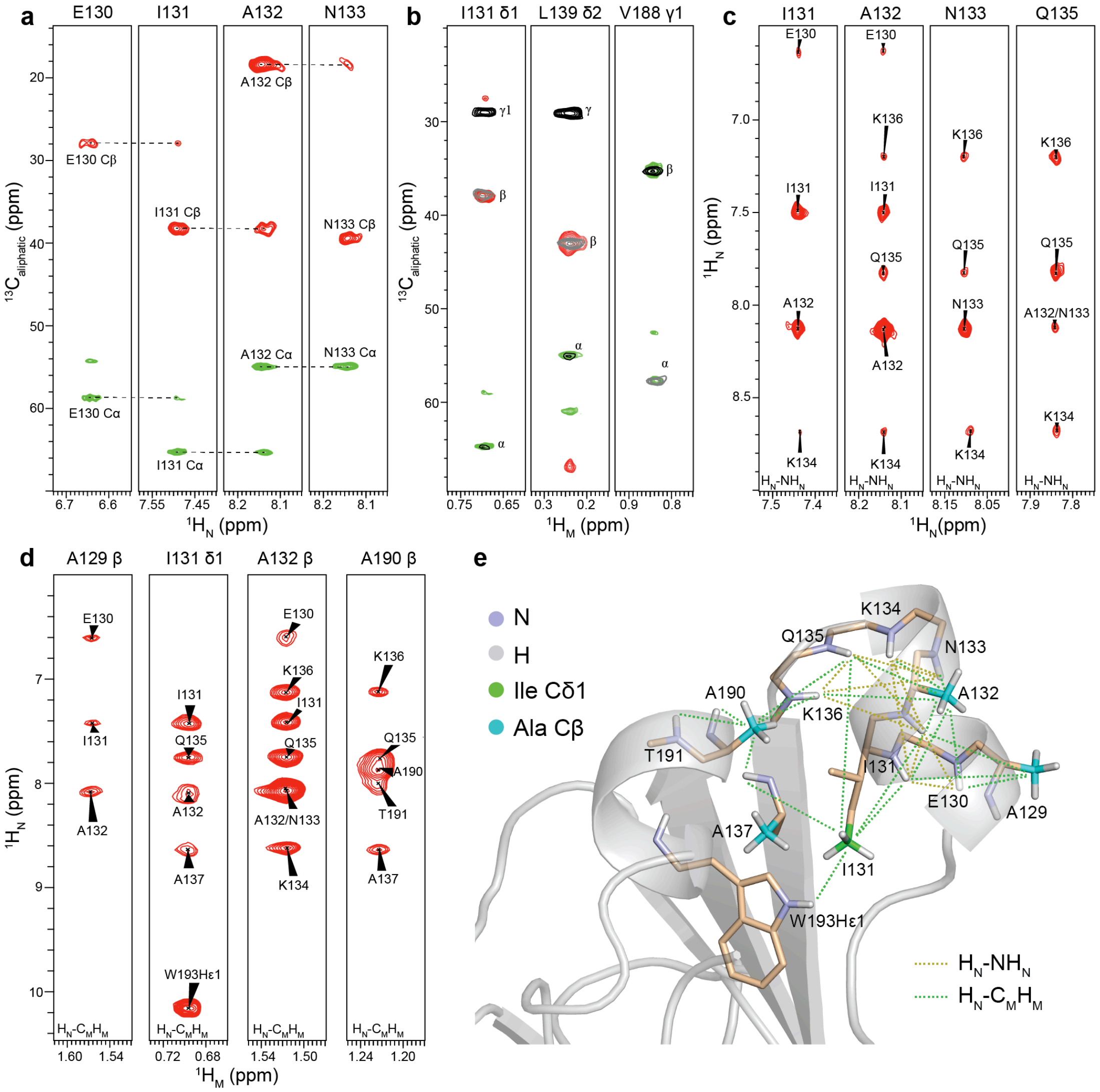

Supplementary Figure 5 | Assignments of residues near the Cβ H3 helix region

a, $^{13}C_{aliphatic}$-$^{1}H_{N}$ strips from 3D HNCA (green) and 3D HN(CA)CB (red) experiments showing sequential assignment of residues E130 to N133 on the H3 helix. **b,** $^{13}C_{aliphatic}$-$^{1}H_{M}$ strips from a 3D HMCM[CG]CBCA experiment showing assignment of sidechain methyls on or near the H3 helix region, including I131 δ1, L139 δ1, and V188 γ1. Negative correlations (^{13}Cβ for Ile and Leu, ^{13}Cα for Val) are shown in grey, while positive correlations (^{13}Cγ1 for Ile, ^{13}Cγ for Leu, ^{13}Cβ for Val) are shown in black. The 3D HNCA (green) and 3D HN(CA)CB (red) strips for each residue are overlaid onto their respective 3D HMCM[CG]CBCA strip. **c,** $^{1}H_{N}$-$^{1}H_{N}$ strips from a 3D H_N-NH_N NOESY experiment showing NOEs between residues on the H3 helix. **d,** $^{1}H_{N}$-$^{1}H_{M}$ strips from a 3D H_N-C_MH_M SOFAST NOESY experiment showing NOEs between residues on the H3 helix. **e,** Close up of the H3 helix region of the β-chain from the X-ray structure of the B4.2.3 TCR showing NOEs between residues in the 3D H_N-NH_N (yellow dotted line) and 3D H_N-C_MH_M (green dotted line) NOESY experiments. Atoms are colored as followed: amide N (light blue stick), amide H (grey stick), Ile δ1 (green stick), and Ala Cβ (cyan stick). Resonance assignments of the p/MHC-bound form of the TCR were transferred from the established free form assignments using the two complementary SOFAST NOE datasets outlined in **c, d**.

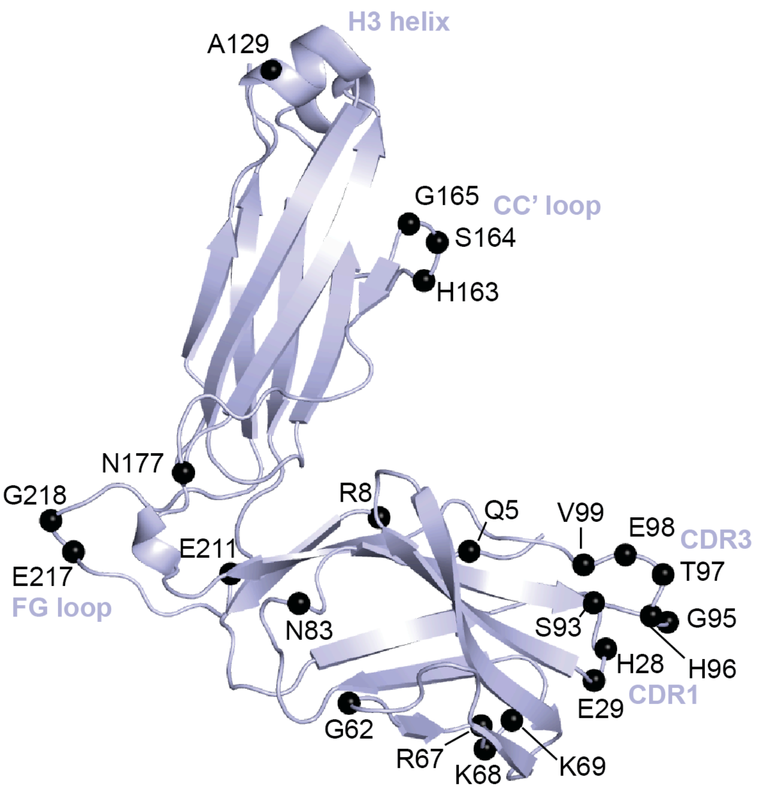

Supplementary Figure 6 | Mapping of β-chain residues with unassigned backbone resonances onto the X-ray structure of the TCR

Using a combination of 3D experiments (outlined in **Supplementary Fig. 2**), 90% of expected backbone amide resonances of β-chain labeled B4.2.3 TCR (total number of residues is 236) were assigned in the 2D ^{1}H-^{15}N TROSY-HSQC spectrum (**Fig. 3a**). Residues lacking backbone amide assignment are mapped onto the X-ray structure of the β-chain of the B4.2.3 TCR, and shown as black spheres. These residues primarily map to loops, including the CC’ loop, FG loop, CDR3 loop, and CDR1 loop. Unassigned residues M1 and K2 are also missing from the X-ray structure and therefore are not shown. In addition, 91 out of 91 (100%) expected Ala ^{13}Cβ, Ile ^{13}Cδ1, Leu ^{13}Cδ1/^{13}Cδ2, Val ^{13}Cγ1/^{13}Cγ2 sidechain methyl chemical shifts were stereo-specifically assigned in the 2D ^{1}H-^{13}C SOFAST HMQC spectrum (**Fig. 6a**) of β-chain labeled B4.2.3 TCR.

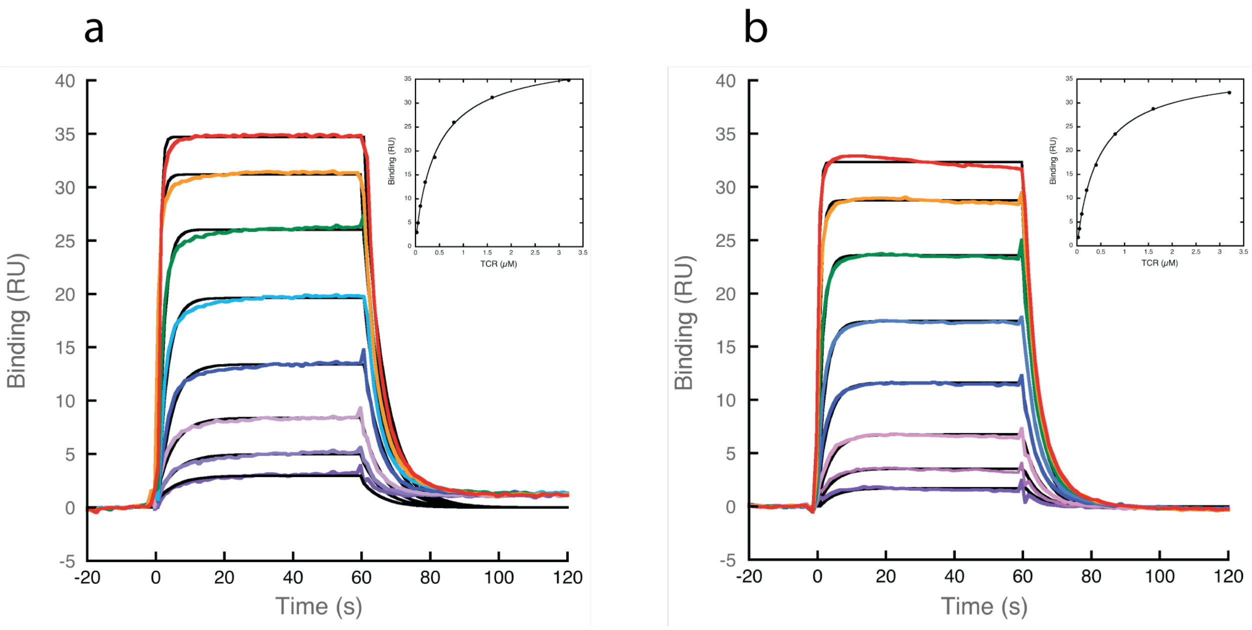

Supplementary Figure 7 | Binding of TCR and Cβ Asn133A mutant to pMHC by SPR
Biotinylated P18-I10/H2-D^{d}/mβ2m complexes were coupled to a streptavidin chip in a BIAcore T200 instrument to 100 resonance units (RU) and purified **a,** B4.2.3 TCR or **b,** Cβ Asn133A mutant TCR were offered in graded concentrations ranging from 25 nM to 3.2 μM. Curves were fit to steady state values (inset) or kinetically to a 1:1 binding model using T200 evaluation software 3.0.

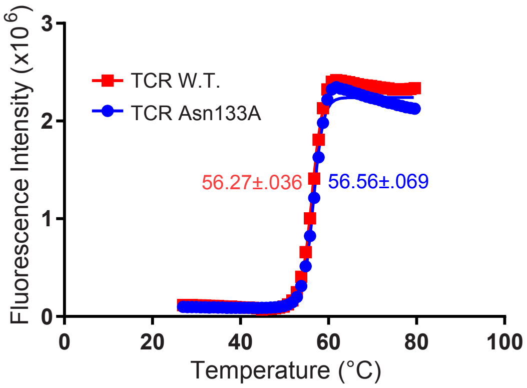

Supplementary Figure 8 | Quantitative thermal stability analysis of TCR and Cβ Asn133A mutant using differential scanning Fluorimetry

Accurate melting data were acquired using a 96 well plate format on an Applied Biosystems ViiA qPCR machine. Individual wells contained a final concentration of 7 µM of each protein sample (in 25 mM Tris buffer, PH 8.0, 150 mM NaCl) and 10X SYPRO orange dye. The wells are progressively heated with a rate of 1°C min^{-1}. The raw intensity data (shown as colored points) were fit with a Boltzmann sigmoidal curve (solid lines) to determine melting temperatures, as indicated for each protein.
